# Extensive Physiotherapy Rehabilitation for Pulmonary Tuberculosis and Tuberculous Meningitis With Ventriculoperitoneal Shunting: A Case Report

**DOI:** 10.7759/cureus.70578

**Published:** 2024-09-30

**Authors:** Samruddhi Aherrao, Pallavi Harjpal, Anshu Tikariha

**Affiliations:** 1 Department of Cardiorespiratory Physiotherapy, Ravi Nair Physiotherapy College, Datta Meghe Institute of Higher Education and Research (Deemed to be University), Wardha, IND; 2 Department of Neuro-Physiotherapy, Ravi Nair Physiotherapy College, Datta Meghe Institute of Higher Education and Research (Deemed to be University), Wardha, IND

**Keywords:** intensive care unit, pulmonary tuberculosis, rehabilitation, sensations, ventriculoperitoneal shunting

## Abstract

Tuberculous meningitis (TBM), an advanced form of extrapulmonary tuberculosis (TB), is caused by *Mycobacterium tuberculosis* infection of the meninges surrounding the brain and spinal cord. If it is not promptly and effectively treated, it can result in serious inflammatory reactions and neurological problems. TBM frequently leads to joint stiffness, discomfort, respiratory difficulties, swallowing difficulties, neurological deficits, and muscular weakness. Physiotherapy intervention is essential in treating these issues, as it provides individualized treatment strategies and programs that enhance muscle strength, motor coordination, and overall mobility. Acute TB is a disease that spreads through the bloodstream due to the presence of TB bacteria. The mild, nonspecific clinical presentation, which typically mimics the central organ involved, makes diagnosis challenging. This case study explains how to enhance function, quality of life, and functional capacity. It examines a 43-year-old man who reports respiratory issues, swallowing difficulties, and generalized weakness. MRI results indicate a defect in the right parietal region, along with slight soft tissue swelling above it. A four-week physical therapy rehabilitation program was created based on the patient's limitations identified during the initial intensive care unit (ICU) phase. The primary objectives of physiotherapy were to promote postural balance, preserve joint integrity, initiate early bed mobility, enhance sensation by improving respiratory patterns and secretion mobilization, and assist the patient with transfers and activities of daily living (ADLs).

## Introduction

Tuberculosis (TB) is an airborne infectious disease that primarily affects the lungs. The microorganism *Mycobacterium tuberculosis *(MTB)* *is responsible for causing TB. If the infection is not treated promptly, the bacteria may spread throughout the bloodstream and harm other tissues and organs [1]. Infection with TB within the central nervous system (CNS) can lead to meningitis, tuberculoma, and spinal arachnoiditis. MTB is the causative agent of tuberculous meningitis (TBM), which is characterized by inflammation of the membranes surrounding the brain or spinal cord [2]. However, the prompt detection of TBM remains a major challenge, as symptoms such as high fever, headache, and vomiting are ambiguous. The diagnosis of TBM generally relies on clinical suspicion and empirical decision-making [3].

Bacterial meningitis is a medical emergency that, when left untreated, may contribute to infection of the blood and brain damage. This is manageable with intravenous antibiotics, and in severe instances, admission to critical care will be necessary to monitor vital signs [4]. The vast majority of patients remain asymptomatic during the initial infection. Most individuals recover from their sickness; however, a section enters a "latent" phase with the potential for "reactivation" in the future. Symptomatic patients (approximately 10%) develop initial lung infections, with some experiencing progression to other organs, especially immune-compromised patients (e.g., HIV patients). The most commonly observed symptom is a persistent fever, but only one-third of patients with pulmonary problems have difficulty breathing [5]. Delays in detecting and treating pulmonary TB (PTB) are the primary causes of death, particularly in patients with acute pulmonary failure. To avoid ICU hospitalization and complications, a quick diagnosis and efficient treatment must be implemented [6]. The spread of Mycobacterium is a potentially fatal infection, particularly if treatment and diagnosis are delayed. Due to the generic clinical picture and the limited number of methods available for confirmatory laboratory diagnosis, including the time-consuming nature of testing, low specificity of the acid-fast bacilli (AFB) smear, and the inability to detect miliary changes in chest X-rays, the diagnosis can be difficult to make [7].

There is a complicated connection between deafness and TBM, where damage to the cortical language area results in a language disability. The Wernicke, Broca, and arcuate fasciculus areas of the brain are responsible for language and speech production [8]. PTB may trigger irreversible lung damage, which is generally observed by radiography as the condition known as fibrosis and cavitation. Chronic loss of lung function is linked to long-term respiratory symptoms and clinical presentations as chronic respiratory diseases (CRDs), such as chronic obstructive pulmonary disease (COPD), bronchiectasis, and other diseases [9]. Pulmonary physiotherapy is suggested as a broad patient-tailored strategy that combines exercise education, instruction, and behavioral change, carried out after a thorough, modified examination. Its goal is to improve the psychological and physical well-being of individuals suffering from persistent respiratory conditions while additionally encouraging long-term participation in health-promoting behaviors [10].

Ventriculoperitoneal (VP) shunt surgery is one of the most prevalent procedures used to treat tuberculous hydrocephalus. There is little information on concerns surrounding the indication and timing of spinal fluid diversion procedures in TBM [11]. Patients with obstructive hydrocephalus or who have failed an endoscopic third ventriculostomy (ETV) may benefit from a VP shunt. This device employs a small elastic tube to drain the spinal fluid that circulates from the brain into the intestines. A valve controls the amount of fluid drained [12]. As part of this interdisciplinary approach, physiotherapy attempts to assess the gait pattern of individuals with normal pressure hydrocephalus (NPH), which appears as an inability to perform a series of motions and a lack of motor and sensory challenges. Depending on the level of complexity, patients may require some external support to walk or may be unable to complete their gait [13].

## Case presentation

Patient information

A 43-year-old female patient visited the Acharya Vinobha Bhave Rural Hospital's Emergency Department in Sawangi, Wardha, India, with chief complaints of headache, fever, blurred vision, and vomiting for 3-4 days, leading to significant weakness. For the last 2-3 days, she had difficulty speaking and also experienced challenges with eating and swallowing. There was a decrease in movement of the bilateral upper limbs and lower limbs, attributed to weakness. For these reasons, she sought medical attention, where a neurological examination was performed. At the time of the examination, the patient was oriented to place, person, and time. The examination revealed normal reflexes on the right side and reduced reflexes on the left side. Tone assessment indicated hypotonia on the left side and normal tone on the right side, using a tone grading scale for evaluation. A sensory examination was also conducted, and cranial nerves were assessed. CT scan and MRI findings revealed a defect located in the right parietal region with minimal overlying soft tissue swelling. Tests for liver and renal function were both within acceptable limits. Human immunodeficiency virus (HIV), hepatitis B surface antigen (HBsAg), and hepatitis C virus (HCV) viral markers were not present. A chest X-ray showed mild consolidation in the right lower zones. The timeline of events is shown in Table 1.

**Table 1:** The events and timeline of the condition

Events	Timeline
History of vomiting, headache, and fever	May 13, 2024
Date of admission	May 17, 2024
Date of investigation	May 18, 2024
Date of surgery (ventriculoperitoneal shunting)	May 20, 2024
Date of physiotherapy rehabilitation	May 21, 2024

Neurological examination

**Figure 1:**
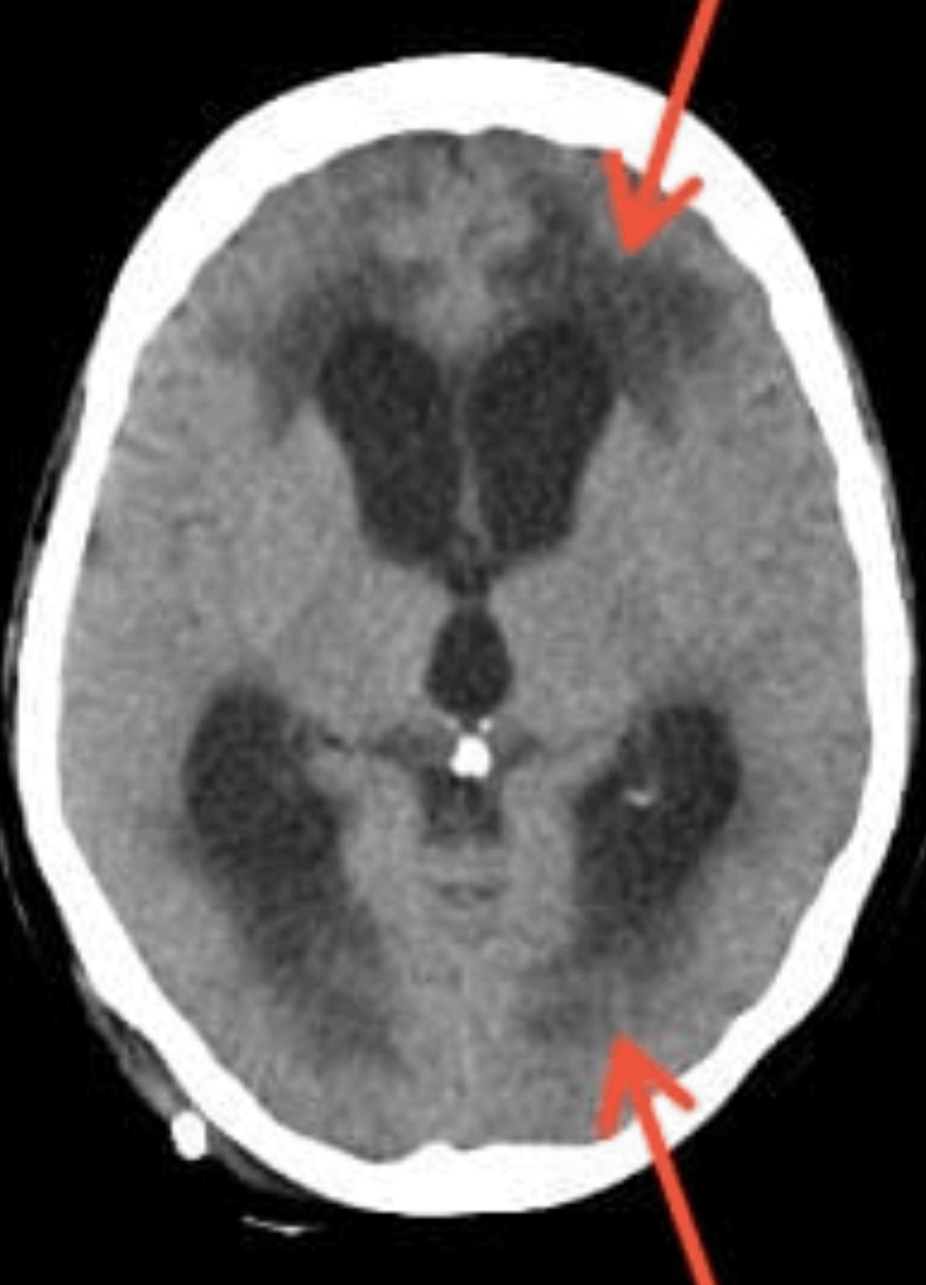
Air foci adjacent to the frontal horn of left lateral ventricle, suggesting pneumocephalus and mild dilation of bilateral lateral ventricles

**Figure 2:**
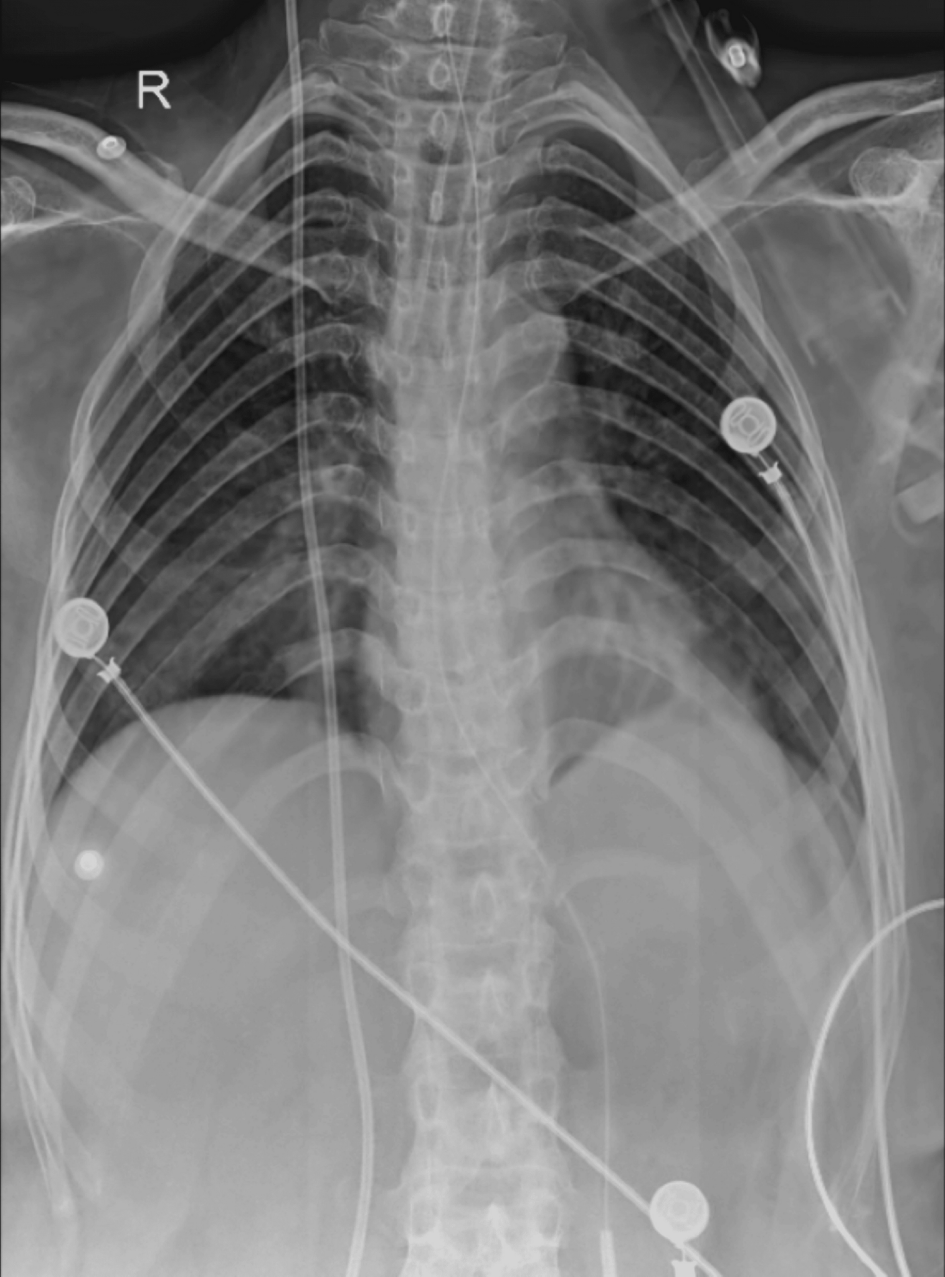
Chest X-ray showing the bronchovesicular markings present in left upper, middle, and lower zones

The first examination was carried out with the patient's and family's consent. The patient was alert, cooperative, and aware of time, location, and person. The patient was in a supine position, and thus the neurological assessment was conducted in the same position. The score on the Glasgow Coma Scale (GCS) was E4V5M6 for higher mental function testing. The patient was connected to external medical devices such as a Foley catheter and a Ryles tube. The sensory examination revealed that sensory functions in the left upper extremity were absent, while the right upper limb sensory functions were intact. However, the lower extremities lacked superficial reflexes, such as light touch and pinprick sensation, on the left side, with intact sensation on the right side. Muscle tone in the left upper limb was hypotonic, while it was normal in the right upper limb and the same for the lower extremities. Based on the Oxford grading system, the right upper limb was graded 3/5 (full range of motion against gravity without resistance), while the left lower limb was graded 2/5. MRI revealed a defect located in the right parietal region with minimal overlying soft tissue swelling and sutures in situ. A VP shunt was in place, with its tip noted in the frontal horn of the right lateral ventricle. Mild dilation of the bilateral lateral ventricles and chronic lacunar infarcts were noted in the bilateral gangliocapsular region. A nasal spur was observed on the right side, along with concha bullosa in the left middle turbinate. Investigations are explained in Figures 1, 2.

Manual muscle testing

A vital component of the physical examination that may provide information concerning neurological deficits is testing muscle strength. This usually takes place as part of an objective assessment of the patient. It is a method for evaluating weakness and aids in distinguishing between true weakness and issues related to imbalance or inadequate endurance. It can be referred to by various synonyms, including manual muscle testing, muscle strength grading, or motor testing. There are several methods for measuring muscle strength, including functional, mechanical, and individual approaches. Testing for muscle strength is shown in Table 2.

**Table 2:** Manual muscle testing (MMT) of bilateral lower limbs

MMT	Pre-treatment		Post-treatment	
Muscles	Right	Left	Right	Left
Shoulder				
Flexors	3/5	2/5	4/5	3/5
Extensors	3/5	2/5	4/5	3/5
Abductors	3/5	2/5	4/5	3/5
Adductors	3/5	2/5	4/5	3/5
Elbow				
Flexors	3/5	2/5	4/5	3/5
Extensors	3/5	2/5	4/5	3/5
Wrist				
Flexors	3/5	2/5	4/5	3/5
Extensors	3/5	2/5	4/5	3/5
Hip				
Flexors	3/5	2/5	4/5	3/5
Extensors	3/5	2/5	4/5	3/5
Abductors	3/5	2/5	4/5	3/5
Knee				
Flexors	3/5	2/5	4/5	3/5
Extensors	3/5	2/5	4/5	3/5
Ankle				
Dorsiflexion	3/5	2/5	4/5	3/5
Plantarflexion	3/5	2/5	4/5	3/5

Sensory examination

A neurological sensory assessment is a clinical evaluation that assesses the sensory functions of the nervous system. It involves a battery of tests to evaluate the condition and functioning of sensory systems, including touch, pain, temperature, vibration, position awareness, and proprioception. Bigley's study provides a summary of the key elements of a neurological sensory examination [14]. The examination is further explained in Table 3.

**Table 3:** Superficial sensory examination

Superficial Sensations on the Upper Limb and the Lower Limb	Left	Right
Pain	Absent	Present
Touch	Absent	Present
Temperature	Absent	Present
Pressure	Absent	Present

Deep sensory examination

Deep sensory examination includes proprioception, kinesthesia, and vibration. Proprioception refers to the sense of position and awareness of a joint while it is at rest. The therapist maintains the joint in a stationary position while allowing it to move through its range of motion. Next, the patient is asked to demonstrate the position with a different limb or to describe it verbally. To evaluate the subject's ability to perceive a vibratory stimulus, the base of a vibrating tuning fork is placed on a bony prominence (such as the sternum, elbow, or ankle). The tuning fork's frequency should generally be 128 Hz. If the patient has any impairment, they will be unable to distinguish between a vibrating and non-vibrating tuning fork. Consequently, it is necessary to apply vibrating and non-vibrating stimuli randomly. This is explained in Table 4.

**Table 4:** Deep sensory examination

Deep Sensations on the Upper Limb and Lower Limb	Left	Right
Proprioception	Absent	Present
Kinesthesia	Absent	Present
Vibration	Absent	Present

Reflexes

In response to a stimulus, reflexes are instinctive movements that occur almost instantly. Reflexes are involuntary reactions to stimuli that happen through a reflex arc and do not require conscious awareness. This is explained in Table 5.

**Table 5:** Reflex assessment +: Diminished, ++: Normal reflex, +++: Brisk reflex, ++++: Exaggerated reflex

Reflexes	Right	Left
Biceps	++	+
Triceps	++	+
Brachioradialis	++	++
Supinator	++	Not assessable (due to IV line)
Knee	++	+
Ankle	++	+
Plantar	Flexor response	Flexor response

Tone assessment

A component of motor evaluation is tone assessment, which pertains to the evaluation of muscular tone. Muscle tone is essential for maintaining posture, facilitating movement, and ensuring fluid and coordinated muscular activity. This is explained in Table 6.

**Table 6:** Tone assessment in bilateral upper and lower limbs according to the tone grading scale

Side	Right	Left
Upper limb	2+	1+
Lower limb	2+	1+

Outcome measures

The Barthel Index, ICU Mobility Scale, and Quality of Life Scale are used to assess the functional mobility and activity level of the patient's condition. This is explained in Table 7.

**Table 7:** Outcome measures taken according to patient condition

Outcome Measure	Pre-treatment	Post-treatment
Barthel Index	0/100	30/100
ICU Mobility Scale	1/10	7/10
Quality of Life Scale	50/112	68/112

Physiotherapy intervention

After obtaining a referral from the neurologist, physiotherapy was initiated. The patient underwent physical therapy rehabilitation six days a week for 30 minutes each day. The protocol to be followed, based on the rehabilitation timeframe, is presented in Table 8. The therapy was provided for a duration of three weeks.

**Table 8:** Interventions according the patients' condition PNF: proprioceptive neuromuscular facilitation, NA: not applicable, PACE: program of assertive community treatment.

Problem	Goals	Intervention	Dosage
Patient and family education	To build up and sustain the family's positive mindset toward patient treatment	The patient along with his family to be well explained about the patient's condition and treatment protocol	NA
Generalized weakness	To increase strength and alleviate symptoms of weakness	Increase the ability of every muscle group (via conditioning exercises). Start with the process of PNF D1, which is the first flexion and extension pattern for both the upper and lower extremities, utilizing the hold-relax technique	10 repetitions × 3 sets
Decreased range of movement and motion	To enhance the range of motion and mobility	Passive range of motion exercise of the left upper limb and lower limb; active range of motion exercise of the right upper limb and lower limb	10 repetitions × 2 sets
Reduced sensation	To Improve sensory integration of the left upper and lower limb	Tactile stimulation is given using textured materials like brushes or clothes on the left upper limb and lower limb; thus, it can provide tactile stimulation	3 times a day × 2 repetitions
Difficulty in interpreting speech	To improve articulation, tone, and melody of speech	Speech and language therapy and transcranial magnetic stimulation (20)	For roughly 30 to 60 minutes a day, two times a week, for six weeks),
Auditory and visual impairment	To improve auditory and visual feedback	Multimodal stimulation therapy	3 times a day × 2 repetitions
Excessive secretions	To reduce and clear secretions	Oral suctioning is performed, and chest physiotherapy is given with percussion, vibration, and shaking	10 repetitions × 2 sets
Decrease in lung compliance	To improve lung function	End expiratory stretch, diaphragmatic breathing exercises	10 repetitions × 2 sets 10 repetitions × 1 set
Reduce bed mobility	To improve bed mobility and function mobility	Mobility training involves bed rolling, bedside sitting, and wheelchair mobility exercises to encourage functional independence	Thrice a day
Reduce balance and coordination	To improve balance and coordination	Balance training includes a 30-minute elevation of the bed head to test balance and stability. In week 2, keep this routine while integrating more complex balance exercises, such as single-leg stance with support and without support	Twice a day
Lack of effective communication skills	To improve the ability to communicate	PACE therapy	Twice a day
Reduced functional mobility	To improve gait training	Gait training should be initiated with support and as the patients adapt to it, it should be given without support	2–3 rounds a day

## Discussion

A granulomatous inflammation of the basal meninges that progresses slowly is the hallmark of TB. If treatment is not received, this inflammatory response may lead to hydrocephalus, cerebral arterial infarction, cervical nerve palsy, and possibly death. Early detection and treatment are crucial for preventing disease-related death and suffering [15]. The patient in this case study has TBM meningitis and PTB and is receiving VP shunting. Therefore, physiotherapy is essential for restoring the patient to a functional level.

An immunological reaction often results in neurological deficits and muscle weakness in TBM. Physiotherapy interventions are valuable for enhancing muscle strength and range of motion. Through specific exercises, such as passive range of motion and active assisted range of motion, the patient can regain both cognitive function and muscle strength. Physiotherapy helps reverse muscular atrophy and promote coordinated muscle activity, counteracting the muscle weakness associated with TBM. Additionally, physiotherapy effectively addresses respiratory problems that may arise in TBM patients due to compromised respiratory muscles and decreased lung function.

In a study of two cases of severe TBM, the authors emphasized the importance of a comprehensive rehabilitation program that included therapist-based multimodal sensory stimulation for patients in intensive care unit (ICU) settings [2]. Worldwide, TB continues to be a leading cause of disease and death. The severe effects on the patient's life include social, mental, and physical degradation during active TB and post-TB sequelae. Even when specific antituberculous treatment is administered to sputum-negative patients, extrapulmonary symptoms of TB, such as depression and cachexia-including muscle weakness-can persist for a long time [16]. In high-burden settings, PTB leads to significant, long-term impairments in quality of life, exercise tolerance, and lung function. Current estimates of the disease burden likely underestimate its full impact. Early diagnosis and treatments that modify the condition could lessen the long-term consequences of PTB [17].

In this study, patients with chronic NPH had their cognitive function and activities of daily living compared. One group received a VP shunt, while the other did not. Findings indicated that VP shunt therapy, in addition to rehabilitation, was more effective than rehabilitation alone [18]. Nearly half of the patients with functional deficits in this trial by de la Mora et al. had central airway obstruction (CAO) at the conclusion of PTB treatment. PTB sequelae led to significant fixed CAO and negatively impacted quality of life, even though these patients were considerably younger than those with smoking-related COPD [19]. The focus on intensive early rehabilitation, family education, and ongoing collaboration between medical and rehabilitation specialists makes this formalized approach unique [20].

The primary role of this case report is to improve strength, bed mobility, and sensation while reducing cardiovascular and pulmonary complications through chest physiotherapy and breathing exercises. The physiotherapist presented a comprehensive physical therapy rehabilitation program that included various exercises and resistance equipment. Passive range of motion, active assisted range of motion, multimodal sensory stimulation, and sensory integration aim to improve functional skills and daily living activities.

## Conclusions

In this case study, a 43-year-old woman underwent VP shunting due to TBM and PTB. Consequently, physical therapy played a crucial role in her recovery by facilitating her return to functional activity, strengthening and enhancing sensory function, and alleviating chest issues. This comprehensive rehabilitation approach addressed accompanying physical infirmities and functional restrictions, as well as cognitive challenges, thereby improving her mobility, speech articulation, comprehension, and overall verbal efficiency. Ultimately, physiotherapy supported the patient in enhancing her daily activities and quality of life.
